# Evaluation of mesalazine polymeric conjugate in the treatment of actinic proctitis in rats

**DOI:** 10.1590/ACB360805

**Published:** 2021-10-08

**Authors:** Vinícius Rodrigues Taranto Nunes, Paula Vieira Teixeira Vidigal, Márcio Tadeu Pereira, Luís Carlos Duarte Ladeira, Marcelo Vidigal Caliari, Fabrício Marcus Silva Oliveira, Aina Liz Alves Cesar, André Augusto Gomes Faraco, Rafael Calvão Barbuto, Ivana Duval-Araujo

**Affiliations:** 1 MD, PhD. Medical School - Universidade Federal de Minas Gerais (UFMG) – Belo Horizonte (MG), Brazil.; 2 PhD, Associate Professor. Medical School - Universidade Federal de Minas Gerais (UFMG) – Belo Horizonte (MG), Brazil.; 3 Nuclear Physicist, PhD. Centro de Desenvolvimento da Tecnologia Nuclear (CDTN) – Belo Horizonte (MG), Brazil.; 4 PhD, Associate Professor. Department of General Pathology - Institute of Biological Sciences - Universidade Federal de Minas Gerais (UFMG) – Belo Horizonte (MG), Brazil.; 5 PhD. Laboratory of Immunology and Parasite Genomics - Department of Parasitology - Institute of Biological Sciences - Universidade Federal de Minas Gerais (UFMG) – Belo Horizonte (MG), Brazil.; 6 Fellow PhD degree. Postgraduate Program in Pharmaceutical Sciences. Department of Pharmaceutical Products - Universidade Federal de Minas Gerais (UFMG) – Belo Horizonte (MG), Brazil.; 7 PhD, Associate Professor. Department of Pharmaceutical Products - Universidade Federal de Minas Gerais (UFMG) – Belo Horizonte (MG), Brazil.

**Keywords:** Radiotherapy, Proctitis, Mesalamine, Chondroitin, Inflammation, Rats

## Abstract

**Purpose:**

The present study aimed at testing a new formulation of mesalazine linked to chondroitin sulfate and its components alone in the treatment of actinic proctitis in rats.

**Methods:**

Forty-seven female Wistar rats were submitted to pelvic radiation and divided into eight groups: control A, mesalazine A, chondroitin A, and conjugate A, gavage of the according substance two weeks after irradiation and sacrifice three weeks after oral treatment; control C, mesalazine C, chondroitin C, and conjugate C, sacrifice six weeks after oral treatment. The rectum was submitted to histological characterization for each of the findings: inflammatory infiltrate, epithelial degeneration, mucosal necrosis, and fibrosis.

**Results:**

The inflammatory infiltrate was more intense in chondroitin A, mesalazine A, and conjugate C. The collagen deposition was less intense in chondroitin A, and mesalazine A, and more intense in control C.

**Conclusions:**

Mesalazine and chondroitin alone were efficacious in inducing a delayed inflammatory response, hence reducing the late fibrosis. The conjugate was able to induce an ever more delayed inflammatory response.

## Introduction

Radiotherapy plays a vital role in the management of pelvic malignancies, especially in rectal tumors. However, its usage implies in several injuries to the surrounding organs, and this damage accompanies patients throughout their lives. As more patients receive radiotherapy as part of their cancer treatment, and ensure a higher rate of survival, the incidence of radiotherapy complications continues to increase^1^.

Radiation to the pelvis, when in contact with the rectum and distal sigmoid colon, can induce actinic proctitis, an incurable injury which can lead to severe gastrointestinal toxicity.

The severity of the induced injury depends on the duration of exposure, the period of time from the radiation, the total radiation dose, the fractioned radiation dose, the radiation dose rate and also some individual factors. During radiotherapy treatment and a few weeks later, the intestinal mucosa is the most damaged layer, in a phase named acute proctitis^2^. Such phase is characterized microscopically by epithelial ulceration and mucosal and submucosal inflammation^3^. After a few months or years, all the layers can be damaged, determining chronic proctitis, which is characterized by excessive extracellular matrix deposition, vascular sclerosis, and muscular dystrophy^3^.

About half the patients submitted to radiotherapy report that their quality of life is affected by gastrointestinal symptoms, which include rectal bleeding, urgency, constipation, tenesmus, diarrhea and rectal pain^4^. In order to minimize those symptoms and treat actinic proctitis, there are some pharmacological options, such as 5-aminosalicylic acid (5-ASA) – mesalazine –, corticosteroids, sucralfate (oral and enemas), formalin, short-chain fatty acids, among others^5^,^6^. In literature, this pharmacological arsenal has controversial results, and gold-standard treatment is not yet well defined.

Among the drugs available so far, mesalazine is one of the most studied, mainly due to favorable therapeutic response in inflammatory bowel diseases, such as Crohn’s disease or ulcerative colitis. Mesalazine inhibits cycloxygenases (COX), COX 1 and 2, inhibiting therefore the synthesis of inflammatory prostanoids, and has been proven promising for treating actinic proctitis in some studies^7^. Nonetheless, its inconvenient dosing scheme impairs the adherence to treatment and makes results even more questionable.

The development of a new formulation of mesalazine that increases the release time of drug action was recently published and proven effective in vitro^8^. Cesar *et al*.^8^ reported the development and characterization of the polymeric prodrug obtained through the linkage of chondroitin sulfate with 5-ASA, corresponding to a novel molecule. Chondroitin sulfate is a biodegradable, biocompatible and mucoadhesive sulfated glycosaminoglycan^9^. It is approved by the Food and Drug Administration (FDA) and widely used as a dietary supplement for osteoarthritis, with anti-inflammatory effect^10^.

Studies involving radiation and its effects in animal’s experimental models are vast in the literature, mostly trying to determine drugs and means of preventing and treating the damage caused by radiation^11^-^13^. Most of the research available is based on radiation given by teletherapy machines. Our research group previously described a new method for inducing experimental actinic proctitis using a natural source of radiation in a nuclear technology research center (Centro de Desenvolvimento da Tecnologia Nuclear), which has proven effective and feasible^14^.

In this work, the efficacy of this new recently developed drug in the treatment of actinic proctitis in rats was evaluated. This is the first in-vivo trial to determine its real effect.

## Methods

### Subjects and experimental groups

The study was approved by the Research Ethics Committee of the Universidade Federal de Minas Gerais (CEUA UFMG Project No. 139/2016).

Forty-seven female Wistar rats weighing around220-280 g and aged 2-3 months old were used in this study, obtained from the Universidade Federal de Minas Gerais (UFMG). The rats were housed in polycarbonate cages (49 × 34 × 16 cm), with four individuals/cage, under controlled conditions (temperature, humidity, air flux). Throughout the experimental period, all mice had access to food (Purina Lab, Curitiba, PR, Brazil) and filtered water *ad libitum*.

The animals were divided into eight groups, according to the solution used (control, mesalazine, conjugate and chondroitin) and the periods of evaluation, acute (A, analysis after three weeks from the oral treatment), and chronic (C, analysis after six weeks from the oral treatment), as follows:

- Control A (n=6): subjected to pelvic irradiation at time zero, placebo (saline 0,9% solution) gavage two weeks after irradiation and sacrifice three weeks after oral treatment;
- Control C (n=5): subjected to pelvic irradiation at time zero, placebo (saline 0,9% solution) gavage two weeks after irradiation and sacrifice six weeks after oral treatment;
- Mesalazine A (n=6): subjected to pelvic irradiation at time zero, mesalazine gavage two weeks after irradiation and sacrifice three weeks after oral treatment;
- Mesalazine C (n=6): subjected to pelvic irradiation at time zero, mesalazine gavage two weeks after irradiation and sacrifice six weeks after oral treatment;
- Conjugate A (n=6): subjected to pelvic irradiation at time zero, mesalazine polymeric conjugate gavage two weeks after irradiation and sacrifice three weeks after oral treatment;
- Conjugate C (n=6): subjected to pelvic irradiation at time zero, mesalazine polymeric conjugate gavage two weeks after irradiation and sacrifice six weeks after oral treatment;
- Chondroitin A (n=6): subjected to pelvic irradiation at time zero, chondroitin gavage two weeks after irradiation and sacrifice three weeks after oral treatment;
- Chondroitin C (n=6): subjected to pelvic irradiation at time zero, chondroitin gavage two weeks after irradiation and sacrifice six weeks after oral treatment.

### Pelvic irradiation

**Figure 1:**
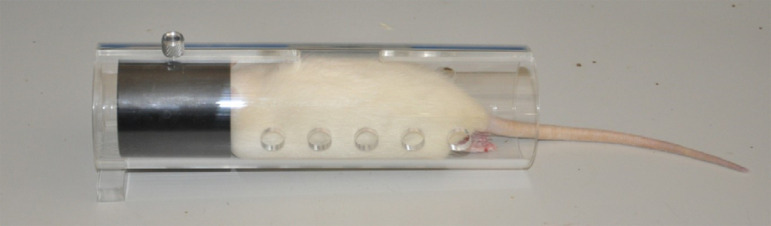
Rat anesthetized placed in the acrylic cylindrical compartment.

**Figure 2:**
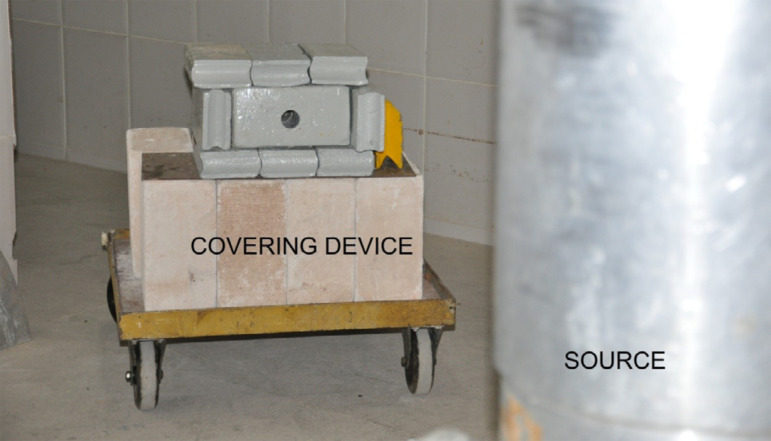
Protection plumb device and collimator around the source.

**Figure 3:**
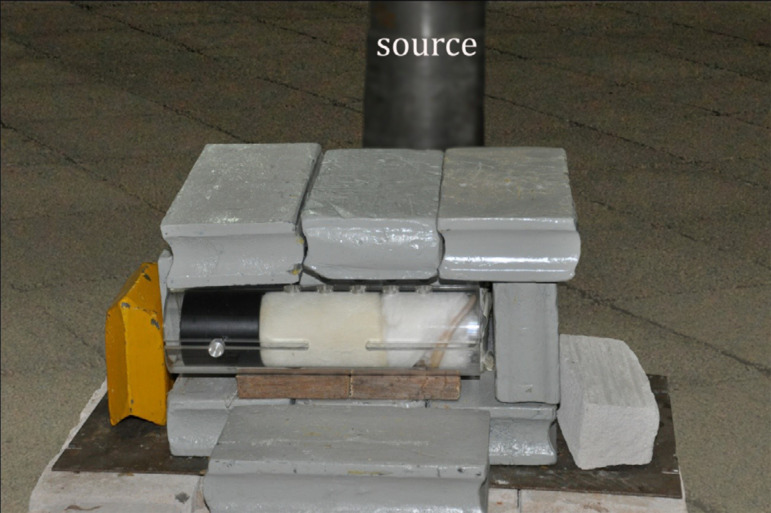
Rat protected with a covering device at 2 m from the source.

All experimental procedures were performed on anesthetized rats. Anesthesia was maintained with ketamine and xylazine (60 and 8 mg/kg i.p.). After properly anesthetized, the rats were set in an acrylic cylindrical compartment (Fig. 1). They were transferred into a protected room which has a Cobalt-60 source in the center. A covering device (15-cm thick plumb bricks with a 32-mm round opening in the center to provide a collimator) was used to protect the rest of the animal’s body from the radiation shade (Fig. 2). The total radiation single dose was 10 Gy. The animals were set in the device which was placed 2 m away from the source (Fig. 3). The radiation dose rate was 76.38 Gy/h, and the animals were irradiated for 7 minutes and 51 seconds in order to achieve the 10 Gy total dose. In the date of the irradiation, the source had the activity of 1.3 × 1,015 Bq.

### Actinic proctitis treatment: gavage

In order to treat the induced actinic proctitis, four modalities of treatment were proposed:

- 0,9% saline solution (groups control A and C) daily for six consecutive days;
- mesalazine (mesalazine A and C) daily for six consecutive days;
- mesalazine polymeric conjugate (conjugate A and C) in three doses, every other day;
- chondroitin (chondroitin A and C) in three doses, every other day.

All substances were diluted for 1 mL of the solution and were administered through gavage. The dose of the mesalazine administered was 88 mg/kg/dose. The dose of chondroitin was 1.05 g/kg/dose. The dose of the conjugate was 88 mg/kg/dose.

### Necropsy

At the end of the observation period (five or eight weeks, according to each group), the animals were submitted to euthanasia by anesthetic overdose (intraperitoneal 200 mg/kgketamine + 25 mg/kg xylazine), and a 2-cm segment of the rectum just above the peritoneal reflection was obtained through laparotomy, and fixed in 10% buffered formalin.

### Histological analysis

The rectum was fixed in 10% buffered formalin, dehydrated in successive solutions of alcohols, and embedded in paraffin. The paraffin blocks were sectioned in a microtome, obtaining 5-?m sections, which later were stained with hematoxylin/eosin (HE) and trichrome stain for histopathological analysis. Slides were examined by the same pathologist under a light microscope (Nikon Eclipse E600), twice, in a blinded manner. Each specimen was subjectively graded for each of the histological findings: inflammatory infiltrate in the lamina propria, epithelial degeneration, mucosal necrosis for the HE slide, and collagen deposition to the lamina propria for the trichrome slide. For the inflammatory infiltrate and epithelial degeneration, specimens were graded as:

- (0): absent;
- (+): mild thickening in the mucosa, mild glandular distortion and less mucosal inflammation with less infiltration of the crypt epithelia by the inflammatory cells;
- (++): moderate inflammatory infiltrate in the lamina propria, moderate glandular distortion with marked crypt abscesses;
- (+++): severe mucosal inflammation infiltrating the crypt epithelia leading to cryptitis and severe crypt abscesses, with severe gland distortion, epithelial cells revealing severe reactive/regenerative atypia with nuclear widening, hyperchromasia, irregularity pleomorphism with prominent nucleoli and loss of cytoplasmic mucin.

As for the mucosal necrosis and collagen deposition, specimens were graded as (0) absent and (+) present.

### Statistical analysis

Statistical analysis was performed using the Fisher’s exact test, with p < 0.05 accepted as statistically significant.

## Results

### Five weeks after radiotherapy: groups A

No significant difference was observed neither in the epithelial degeneration nor in the mucosal necrosis among groups. The inflammatory infiltrate was more intense in the chondroitin A group (p=0.002) and mesalazine A group (p=0.01) compared to the control A group, but no difference was observed for the conjugate A group. However, the collagen deposition was less intense in the chondroitin A group (p=0.002) and mesalazine A group (p=0.01) compared to the control A group, and no difference was observed for the conjugate A group (Table 1).

**Table 1:** Histological parameters five weeks after radiotherapy: groups A.

	Inflammation		Degeneration		Necrosis		Fibroplasia	
	n	%	n	%	n	%	n	%
Control A (n=6)	0	0^a^,^b^	1	17	1	17	6	100^c^,^d^
Chondroitin A (n=6)	6	100^a^	5	83	4	67	0	0^c^
Mesalazine A (n=6)	5	83^b^	4	67	4	67	1	17^d^
Conjugate A (n=6)	2	33	2	33	2	33	3	50

a p=0.002;

b p=0.01;

c p=0.002;

d p=0.01.

### Eight weeks after radiotherapy: groups C

No significant difference was observed neither in the epithelial degeneration nor in the mucosal necrosis among groups. The inflammatory infiltrate was more intense in the conjugate C group compared to the control C group (p=0,002), the chondroitin C group (p=0.002) and the mesalazine C group (p=0.01). The collagen deposition was more intense in the control C group compared to the chondroitin C group (p=0.01) and mesalazine C group (p=0.002), and no difference was observed for the conjugate C group (Table 2). When comparing the groups A to the groups C, no significant difference was observed neither in the groups nor to any of the analyzed parameters.

**Table 2:** Histological parameters eight weeks after radiotherapy: groups C.

	Inflammation		Degeneration		Necrosis		Fibroplasia	
	n	%	n	%	n	%	n	%
Control C (n=5)	0	0^a^	2	40	2	40	5	100^d^,^e^
Chondroitin C (n=6)	3	50^b^	5	83	4	67	1	17d
Mesalazine C(n=6)	1	17^c^	4	67	3	50	0	0^e^
Conjugate C (n=6)	6	100^a^,^b^,^c^	5	83	5	83	3	50

a p=0.002;

b p=0.002;

c p=0.01;

d p=0.01;

e p=0.002.

## Discussion

With the development of new modern therapies to cure cancer, more and more patients survive tumors and have to deal with the sequelae caused by the treatment^15^. Actinic proctitis is a major aftereffect of radiotherapy and usually impairs quality of life. Currently, patients are not only worried about their cure, but with their quality of life. Therefore, new modalities of radiotherapy (conformational and stereotactic body radiation therapy for instance) are in constant development in order to prevent secondary injuries^16^. Also, new therapeutic strategies have been tested to treat those injuries when they develop.

To test new modalities of treatment, animal models are widely used as they simulate human pathologies with a very reliable similarity^17^. Recently, a new technique for pelvic radiation in rats was developed^14^, and this was one of the first experiment in which this novel approach was used. It proved efficacious as the expected histological parameters were determined in the rats as they would have been in humans with actinic proctitis.

The pathological changes noted after applying radiation to the intestine can be divided into acute and chronic changes. The first pathological changes include inflammatory infiltrate in the lamina propria, microscopic damage to mucosal epithelial cells, and vascular endothelial cells. These changes manifest as marked submucosal edema and cryptitis or crypt abscesses, which characterizes acute actinic proctitis. The chronic effects manifest as progressive fibrosis leading to mucosal atrophy, stricture formation and thrombosis, causing secondary ischemic damage. Radiation proctitis in the chronic phase demonstrates a very significant crypt distortion, vascular telangiectasia, and fibrosis of the lamina propria. Progressive endarteritis appears to be the central mechanism by which the chronic actinic proctitis occur^17^-^19^. It was chosen to evaluate the inflammatory infiltrate in the lamina propria, epithelial degeneration and mucosal necrosis as parameters of the acute proctitis and collagen deposition to the lamina propria as a parameter of the chronic proctitis, since they are easier to evaluate and reproduce, what makes an examiner bias less likely.

Among the therapeutic arsenal for actinic proctitis, mesalazine is the most tested in experiments. It is mainly used for inflammatory bowel disease, but it is also studied (although with questionable results in literature) for actinic proctitis^20^ and diverticular disease^21^. The 5-ASA is a potent inhibitor of the synthesis and release of pro-inflammatory mediators (e.g., nitric oxide, leukotrienes, thromboxanes, and platelet activating factor) and also inhibits the function of several cells implicated in the acute inflammatory and immune response (e.g., natural killer cells, mast cells, neutrophils, mucosal lymphocytes, and macrophages)^22^. Literature is controversial as to mesalazine’s effect in radiation proctitis, some studies showing a beneficial improvement of symptoms^7^ and others revealing even worsening of the symptoms^23^.

The new mesalamine polymeric conjugate for controlled release developed by Cesar *et al.* 8 is a novel promising drug that enables an increased bioavailability, a higher mucoadhesiveness, an easier dosing scheme, and a higher treatment adherence. The mucoadhesiveness capacity allows the reduction of the dose required to obtain therapeutic effect^24^. Consequently, it leads to the reduction of adverse effects, suggesting an increase in treatment adherence^25^. Chondroitin was used in the linkage with 5-ASA in order to create this novel molecule, a polymeric prodrug. Although chondroitin’s spread usage is an adjuvant for the treatment of osteoarthritis, some studies have proven that it is also efficacious for the treatment of radiation cystitis^26^. The exact mechanism is still unknown, but it is clear that it repairs the defect in the glucosamina-glycane coat in the bladder^27^.

In the five-week subgroups, which corresponded mostly to acute changes in the rectal wall, the results proven controversial to a careless observer, as the chondroitin and mesalazine groups presented higher intensity of inflammation and lower intensity of fibroplasia. No difference was noted in the conjugate group compared to the others. Nonetheless, such effect could be explained and understood as the eight-week subgroups were examined. In this period of observation, which represents mostly chronic alterations in the rectum, a reverse result was noted in the inflammatory response, as the conjugate group presented higher inflammation as compared to the others. That indicates that the chondroitin and mesalazine induced a delayed inflammatory response as compared to the control group, and the conjugate probably induced an even more delayed inflammatory peak when administered in this specific posology.

Among the works previously reported, and to the best of our knowledge, there is no report in the literature of a study using chondroitin in the treatment of actinic proctitis. As it is efficacious in the treatment of actinic cystits^28^, it may also be of value in the treatment of actinic proctitis, because it had the same response as mesalazine in all the parameters studied. Further studies are needed in the specific purpose of evaluating the effect of chondroitin in the treatment of actinic proctitis, along with the real mechanism related to this process.

Valid data particularly on the treatment of chronic radiation proctitis are lacking^29^. Nonetheless, in this study mesalazine was effective (when administered in this specific posology) in the treatment of both acute and chronic proctitis. It retarded the inflammatory process (inducing a late inflammation peak when compared to control group) and reduced the fibrosis related to radiotherapy. Such benefit of 5-ASA was also proven by Linard *et al.* 30 as they concluded that mesalazine prevented irradiation-induced inflammatory processes as well as the expression of many pro-inflammatory cytokines. Further studies evaluating the molecular events and cytokines expression when mesalazine is used for the treatment of actinic proctitis are needed.

In the eight-week subgroups, mostly related to chronic changes in the rectum, the chondroitin and mesalazine groups presented lower intensity of fibroplasia. It is also in accordance with the previous results in which a later reduction in fibroplasia is the desired final effect in the treatment of actinic proctitis.

The conjugate group was significantly different from others only in the inflammation parameter after eight-weeks of irradiation. It presented higher intensity of inflammation in this period of evaluation, which determined that the conjugate in the dose used for this study could delay the inflammatory process even further than mesalazine by itself. No difference was found in the acute evaluation, because at this period the conjugate was able to restrain the inflammatory peak and also the fibrosis. In the chronic phase, the conjugate group could no longer restrain the inflammatory peak due to suspension of the drug and long-term effect of irradiation, so the peak was induced at this period. No difference was observed in terms of fibrosis after eight weeks of irradiation, because the period of time of observation was probably not enough to verify this peak or because it really induced an overall reduction in fibroplasia. Further studies with longer periods of observation are needed to elucidate the real effect of the conjugate in long-term fibrosis of the rectal wall. Additional works with longer periods of treatment (longer than six-day gavage) are also needed to clarify if the conjugate could not only retard, but also avoid, an inflammatory peak when administered chronically and continuously.

This study has some limitations, as a consequence of being the first of a new developing line of research of the laboratory team. The experimental design of the study was in order to investigate a specific dose of some drugs into a specific period of observation. As the results discussed earlier were obtained, additional drugs posology, period of treatment and period of observation could be used. Some doubts that remained after the study will be clarified in further studies about the subject.

Moreover, a semi-quantitative analysis was used, which can be equivocal sometimes, and there was no attempt to identify the molecular pathways in which the drugs obtained the revealed results. In further tests, molecular changes and cytokine pattern will be possibly identified during each of the periods of examination. Although a blinded evaluation by the pathologist was selected, the evaluator could not be ruled out bias in the study. Nonetheless, the study brings many new possibilities of treatment and research, not only for the aimed drug (conjugate), but also additional results concerning mesalazine and a new open field for researching chondroitin in the treatment of actinic proctitis.

## Conclusions

The present study demonstrated that mesalazine is efficacious in inducing a delayed inflammatory response, hence reducing the late fibrosis in the treatment of actinic proctitis in rats, using a new experimental model described previously. Moreover, it revealed that chondroitin alone had the same result for mesalazine, what is an original unexpected finding. Also, it proved that mesalazine polymeric conjugate was able to induce an even more delayed inflammatory response, but during the period of study the conjugate did not differ from any of the other drugs in terms of fibroplasia.
